# Waarnemingen.be – Plant occurrences in Flanders and the Brussels Capital Region, Belgium

**DOI:** 10.3897/phytokeys.85.14925

**Published:** 2017-08-08

**Authors:** Roosmarijn Steeman, Wouter Vanreusel, Rutger Barendse, Filip Verloove, Nico Wysmantel, Walter Van den Bussche, Thomas Gyselinck, Pieter Hendrickx, Arnout Zwaenepoel, Pierre Van Vooren, Steven Jacobs, Peter Desmet, Karin Gielen, Marc Herremans, Kristijn R.R. Swinnen

**Affiliations:** 1 Natuurpunt Studie, Coxiestraat 11, 2800 Mechelen, Belgium; 2 Plantenwerkgroep Genk, 3600 Genk, Belgium; 3 Botanic Garden Meise, Bouchout Domain, Nieuwelaan 38, 1860 Meise, Belgium; 4 Natuurpunt, Coxiestraat 11, 2800 Mechelen, Belgium; 5 Natuurpunt Beheer, Coxiestraat 11, 2800 Mechelen, Belgium; 6 WVI, Baron Ruzettelaan 35, 8310 Brugge, Belgium; 7 Ecosystem Management Group, Biology Department, University of Antwerp, Campus Drie Eiken, Universiteitsplein 1, 2610 Wilrijk, Belgium; 8 Research Institute for Nature and Forest (INBO), Kliniekstraat 25, 1070, Brussels, Belgium; 9 Evolutionary Ecology Group, Biology Department, University of Antwerp, Campus Drie Eiken, Universiteitsplein 1, 2610 Wilrijk, Belgium

**Keywords:** native, distribution, observation, citizen science, waarnemingen.be

## Abstract

Waarnemingen.be - Plant occurrences in Flanders and the Brussels Capital Region, Belgium is a species occurrence dataset published by Natuurpunt. The dataset contains almost 1.2 million plant occurrences of 1,222 native vascular plant species, mostly recorded by volunteers (citizen scientists), mainly since 2008. The occurrences are derived from the database http://www.waarnemingen.be, hosted by Stichting Natuurinformatie and managed by the nature conservation NGO Natuurpunt. Together with the datasets Florabank1 (Van Landuyt and Brosens 2017) and the Belgian IFBL (Instituut voor Floristiek van België en Luxemburg) Flora Checklists (Van Landuyt and Noé 2015), the dataset represents the most complete overview of indigenous plants in Flanders and the Brussels Capital Region.

## General description

**Purpose**: Plants have a long history of being recorded by both amateur and professional botanists. Volunteer data from amateur botanists were always an important source of distribution data of plants. The atlas of Flanders and the Brussels Capital region (Van Landuyt et al. 2006) was based on the teamwork of many volunteer botanists, NGOs, scientific institutes and governmental organisations. Since Natuurpunt, the largest nature conservation NGO in Flanders, Belgium, launched the web portal www.waarnemingen.be in 2008, the number of plant observations in Flanders and the Brussels Capital Region has risen sharply. Beside IFBL-mapping and project-related observations, this database is easily used for occasional observations and can be used for monitoring (wildlife) areas. Old notebooks and reports were screened and stored in the database (Steeman et al. 2012). The team of specialized validators motivates the inexperienced observers and validates observations. Here we publish these records on a IFBL (Instituut voor Floristiek van België en Luxemburg) grid cell resolution of 4 × 4 km².

## Data published through

Source publication: http://dataset.inbo.be/planten-natuurpunt-occurrences This paper describes version 1.4 of this resource.

Dataset on GBIF: http://www.gbif.org/dataset/bfc6fe18-77c7-4ede-a555-9207d60d1d86, DOI: https://doi.org/10.15468/fyuklz

**Taxonomic coverage**: the taxonomic reference for the dataset is Heukels’ Flora of the Netherlands by Van der Meijden (2005) which follows the classification as suggested by the Angiosperm Phylogeny Group (APG II 2003).

**General taxonomic coverage description**: The datasets contains 1,222 native vascular plant (Plantae) species (as well as an additional number of subspecies, varieties, forms, hybrids and multispecies) recorded in Flanders and the Brussels Capital Region. This includes angiosperms (flowering plants), gymnosperms, ferns and allies, but not algae, mosses and lichens. If the observer remarked that the specific individual of this native plant was introduced by man, then this is recorded in the field *establishmentMeans*.

## Taxonomic ranks

**Kingdom**: Plantae

**Families**: *Adoxaceae, Alismataceae, Amaranthaceae, Amaryllidaceae, Apiaceae, Apocynaceae, Aquifoliaceae, Araceae, Araliaceae, Asparagaceae, Aspleniaceae, Asteraceae, Athyriaceae, Balsaminaceae, Berberidaceae, Betulaceae, Blechnaceae, Boraginaceae, Brassicaceae, Butomaceae, Buxaceae, Campanulaceae, Cannabaceae, Caprifoliaceae, Caryophyllaceae, Celastraceae, Ceratophyllaceae, Cistaceae, Colchicaceae, Convolvulaceae, Cornaceae, Crassulaceae, Cucurbitaceae, Cupressaceae, Cyperaceae, Cystopteridaceae, Dennstaedtiaceae, Dioscoreaceae, Droseraceae, Dryopteridaceae, Elaeagnaceae, Elatinaceae, Equisetaceae, Ericaceae, Euphorbiaceae, Fabaceae, Fagaceae, Gentianaceae, Geraniaceae, Grossulariaceae, Haloragaceae, Hydrocharitaceae, Hypericaceae, Iridaceae, Juncaceae, Juncaginaceae, Lamiaceae, Lentibulariaceae, Liliaceae, Linaceae, Lycopodiaceae, Lythraceae, Malvaceae, Marsileaceae, Melanthiaceae, Menyanthaceae, Molluginaceae, Montiaceae, Myricaceae, Nartheciaceae, Nymphaeaceae, Oleaceae, Onagraceae, Onocleaceae, Ophioglossaceae, Orchidaceae, Orobanchaceae, Osmundaceae, Oxalidaceae, Papaveraceae, Plantaginaceae, Plumbaginaceae, Poaceae, Polygalaceae, Polygonaceae, Polypodiaceae, Potamogetonaceae, Primulaceae, Ranunculaceae, Resedaceae, Rhamnaceae, Rosaceae, Rubiaceae, Ruppiaceae, Salicaceae, Santalaceae, Sapindaceae, Saxifragaceae, Scrophulariaceae, Solanaceae, Taxaceae, Thelypteridaceae, Typhaceae, Ulmaceae, Urticaceae, Verbenaceae, Violaceae, Zosteraceae*

**Figure 1.**
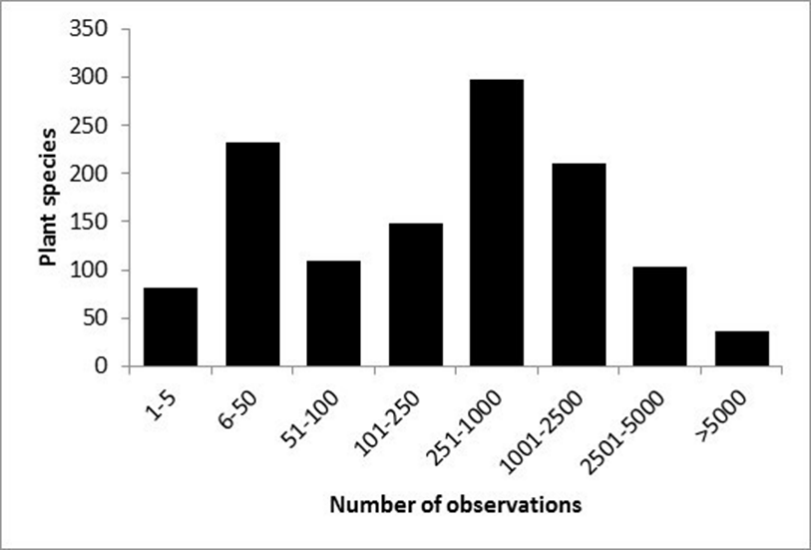
The number of observations per plant species (excluding subspecies, varieties, forms, hybrids and multispecies).

The number of records (observations) per plant species is shown in Fig. 1 and the top 10 most frequently recorded species are shown in Table 1.

**Table 1.** Top 10 of the most frequently recorded plant species in www.waarnemingen.be.

Scientific name	Number of observations
*Urtica dioica*	8687
*Cardamine pratensis*	8446
*Glechoma hederacea*	7741
*Quercus robur*	7695
*Plantago lanceolata*	7438
*Filipendula ulmaria*	7024
*Cirsium arvense*	7010
*Anemone nemorosa*	6902
*Ranunculus repens*	6863
*Achillea millefolium*	6830

## Spatial coverage

**Figure 2.**
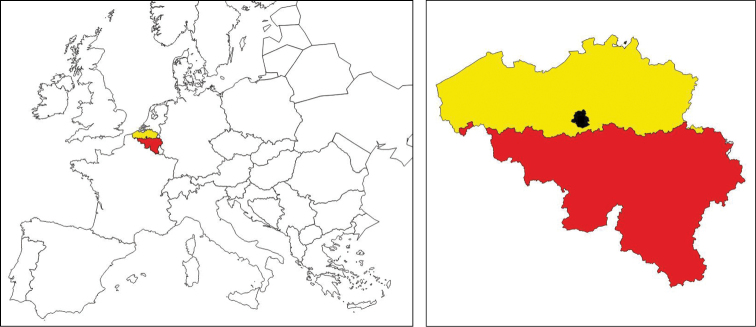
Location of Belgium within Europe (left) and the three administrative regions in Belgium (yellow = Flanders, black = Brussels Capital Region, red = Wallonia)

**General spatial coverage**: Flanders and the Brussels Capital Region (Fig. 2). These regions are situated in the north of Belgium and cover an area of 13,522 km² and 162 km² respectively (13,684 km² in total or 45% of the Belgian territory).

**Figure 3.**
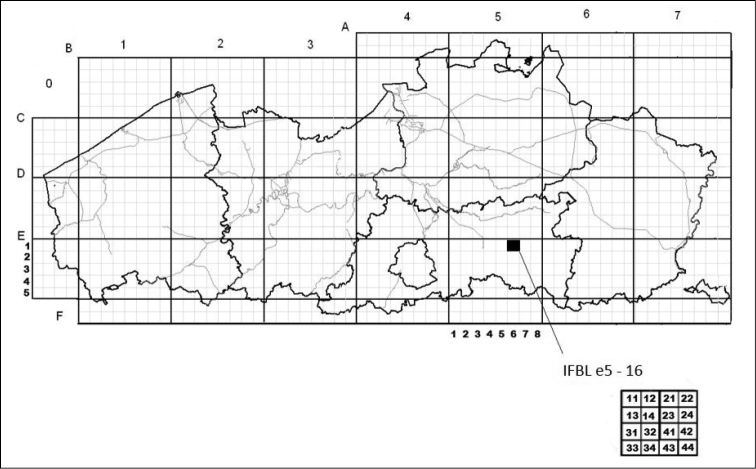
4 × 4 km^2^ IFBL grid cells in Flanders and the Brussels capital region.

Flanders is largely covered by agricultural land (51%), urban areas (30%) and woodland (10%) while the Brussels Capital Region mainly consists of urban areas (73%), woodland (12%) and other green areas (10%) (Vriens et al. 2011). All occurrence data are generalized to IFBL grid cells of 4 × 4 km² (Fig. 3), with the grid codes indicated in the field *verbatimCoordinates*. The WGS84 centroids of these grid cells are calculated in *decimalLatitude/Longitude* with a coordinate *UncertaintyInMeters* of 2,828 meters (using Wieczorek et al. 2004).

**Coordinates**: 50°40'48"N and 51°30'36"N Latitude; 2°32'24"E and 5°55'12"E Longitude.

**Figure 4.**
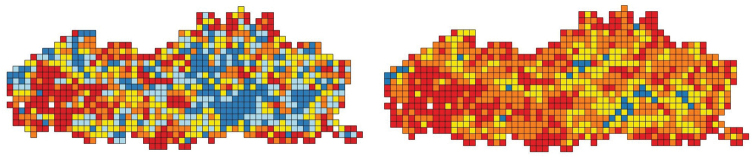
Left: the number of plant observations per IFBL grid cell. Red (1–200), orange (201–500), yellow (501–1000), light blue (1001–2000) and dark blue (2001–14000). Right: the number of plant species (subspecies, varieties, forms, hybrids and multispecies not included) per IFBL grid cell. Red (1–150), orange (151–300), yellow (301–450) and blue (451–600). The two white IFBL grid cells in the west of Flanders are locations without plant observations.

**Figure 5.**
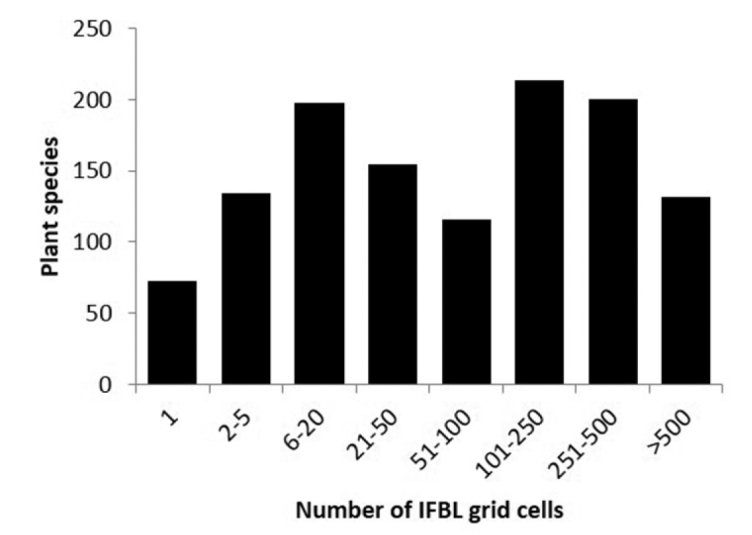
Known distribution based on the data from www.waarnemingen.be of true plant species (subspecies, varieties, forms, hybrids and multispecies not included) based on the number of IFBL grid cells with observation of this species.

We show the number of plant observations and the number of plant species per IFBL grid cell (Fig. 4). Figure 5 shows the frequency distribution of plant species per number of IFBL grid cells. The top 10 of the most widespread recorded plant species is shown in Table 2.

**Table 2.** Top 10 of plant species registered in the most IFBL grid cells.

Scientific name	Number of IFBL grid cells
*Urtica dioica*	790
*Achillea millefolium*	777
*Plantago lanceolata*	772
*Glechoma hederacea*	765
*Ranunculus repens*	765
*Cardamine pratensis*	762
*Cirsium arvense*	743
*Sambucus nigra*	740
*Tanacetum vulgare*	739
*Rumex obtusifolius*	736

**Temporal coverage**: June 30, 1855 - December 31, 2016

**Figure 6.**
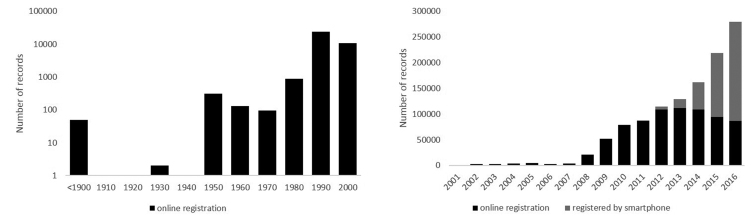
Number of collected records between 1855 and 2000 (left) and between 2001 and 2016 (right). Each number on the left x-axis is a period of 10 year (e.g., 1910 = 1901–1910, etc.). Note the difference between the scales on the y-axis between the left and right figures and the strong increase in smartphone registration of records since the launch of an app (ObsMapp for Android) in 2012.

The majority of records was collected since the launch of www.waarnemingen.be in 2008 (Fig. 6).

## Methods

**Figure 7.**
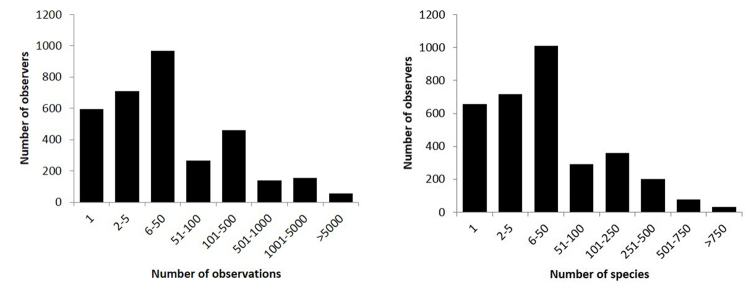
Frequency distribution of observers per number of observations (left) or species (right). Note the difference between the x-axis in the left and right y-axis.

**Sampling description**: Most observations (species, date, location, observer) were recorded by volunteers (citizen scientists). The dataset also includes historical records and datasets imported in waarnemingen.be. The large majority of records (95%) is a casual observation (presence only record). 5% of observations were registered as part of a species checklist. This is also recorded in the field *samplingProtocol.* The frequency distribution of number of observers per number of records or species is shown in Fig. 7.

**Quality control description**: Recorded data are verified by a group of botanical experts (including professional botanists), based on collected specimens, the observer’s species knowledge, added photographs and known species list of locations. The validation procedure from www.waarnemingen.be consists of an interactive procedure in which observers can be asked for additional information by a team of validators, after which the validator manually adds a validation status. Manual validation focuses on rare species, species that are reported outside their known range and observations accompanied by pictures. Records that are not manually validated are additionally checked by an automated validation procedure that takes into account the number of manually validated observations of a species within a specified date and distance range. 12% of the plant records in this dataset are supported by photographs in www.waarnemingen.be. The validation status is indicated in the field *identificationVerificationStatus*, the link to the original record in *references*.

## Datasets

### Dataset description

The Plant occurrences in Flanders and the Brussels Capital Region, is an export from www.waarnemingen.be. The data were standardized to Darwin Core using a SQL query. The included terms are:

occurrenceID, type, language, license rightsHolder, accessRights, references, datasetID, institutionCode, datasetName, basisOfRecord, informationWithheld, dataGeneralizations, individualCount, sex, reproductiveCondition, establishmentMeans, samplingProtocol, eventDate, continent, countryCode, stateProvince, municipality, verbatimCoordinates, verbatimCoordinateSystem, verbatimSRS, decimalLatitude, decimalLongitude, geodeticDatum, coordinateUncertaintyInMeters, georeferenceRemarks, identificationVerificationStatus, taxonID, scientificName, kingdom, taxonRank, scientificNameAuthorship, vernacularName, nomenclaturalCode.

Generalized and/or withheld information: location information is generalized to 4 × 4 km² IFBL grid cells. Observer name, exact XY-coordinates, toponyms, and photographs are not included in the published dataset, but are stored in the source database. The dataset will be updated on GBIF on a regular basis (currently planned every two year).

**Object name**: Waarnemingen.be - Plant occurrences in Flanders and the Brussels Capital Region, Belgium

**Format name**: Darwin Core Archive format

**Format version**: 1.0

**Character encoding**: UTF-8

**Language**: English

**License**: http://creativecommons.org/publicdomain/zero/1.0/

**Usage norms**: http://www.natuurpunt.be/normen-voor-datagebruik

**First publication date**: 2016-12-23

**Distribution**: http://dataset.inbo.be/planten-natuurpunt-occurrences

**DOI**: https://doi.org/10.15468/fyuklz

## Discussion

Since 2010, the number of plant observations registered annually is larger than all the records available in www.waarnemingen.be before 2008. Observations are currently mainly presence only records (95%). Presence is certain, absence of data can have multiple reasons: an IFBL grid cell was not visited, the species was not present/seen, the species was present but not registered in the database. For this reason, since the end of 2016, www.waarnemingen.be focusses more on lists and transect registration. During field work, the route can be tracked via the mobile app ObsMapp. At the end of the excursion, observers can indicate different types of lists, depending on whether: (1) the records are opportunistically collected presence only data (some records of some of the species encountered), (2) all individuals of selected species were registered, (3) all species were recorded or (4) all individuals of all species (more useful for animals than plants). This additional information allows to account for a better observation effort than currently is the case.

The most frequently and widespread observed plant in www.waarnemingen.be is *Urtica dioica*. This species was in Van Landuyt et al. (2006) also the most widespread plant. The other plants on the top 10 of most frequently recorded plants shows there is bias in the data collected by the plant observers of waarnemingen.be. Species like *Poa annua* or *Sagina procumbens* should be seen much more than striking species like *Cardamine pratensis, Filipendula ulmaria* and *Anemone nemorosa*. This might be explained by the observers’ lack of interest in very common species (Mair and Ruete 2016). Furthermore, spatial biases are expected since the data is collected opportunistically without mandatory sampling protocol (Geldmann et al. 2016). Sampling bias related to variation in recorder activity has been grouped in four main categories by Isaac et al. (2014): 1) uneven recording intensity over time, 2) uneven spatial coverage, 3) uneven sampling effort per visit and 4) uneven detectability. We aim to understand these biases better by stimulating the use of species lists rather than the collection of presence only data.
